# Employment of the Albumin to Alkaline Phosphatase Ratio to Predict All-cause Mortality and Cardiovascular and Cerebrovascular Mortality in Adults Aged At Least 60 Years: An Analysis From NHANES 2003–2018

**DOI:** 10.31083/RCM45049

**Published:** 2026-02-06

**Authors:** Zhen Tan, Mei Zhang, Lei Liu, Shuang Li, Xinrui Xue, Yijun Liu, Hongqiang Ren

**Affiliations:** ^1^Department of Cardiology, Suining Central Hospital, 629000 Suining, Sichuan, China

**Keywords:** albumin to alkaline phosphatase ratio, all-cause mortality, cardiovascular and cerebrovascular mortality, adults aged 60 years and above, predictive factor

## Abstract

**Background:**

The relationship between the albumin-to-alkaline phosphatase ratio (AAPR) and all-cause and cardiovascular and cerebrovascular mortalities, in adults aged 60 years and above, remains unclear. Thus, this study aimed to investigate the relationship between the AAPR and all-cause mortality, as well as cardiovascular and cerebrovascular prognosis, in adults aged at least 60 years.

**Methods:**

A total of 13,603 eligible participants were included. Kaplan–Meier curves and log-rank tests were utilized to compare variations in all-cause, cardiovascular, and cerebrovascular mortalities across the AAPR quartiles. Multivariate Cox proportional hazards models and restricted cubic splines (RCS) were applied to examine the associations among the AAPR and all-cause, cardiovascular, and cerebrovascular mortalities.

**Results:**

Cumulative all-cause mortality and cardiovascular and cerebrovascular mortality in the highest AAPR quartile were remarkably lower than in the lowest quartile. A higher AAPR was related to a diminished risk of all-cause mortality [hazard ratio (HR) = 0.64, 95% confidence interval (CI): 0.57–0.71] and cardiovascular and cerebrovascular mortality (HR = 0.73, 95% CI: 0.60–0.90). The AAPR showed a negative linear association with cardiovascular and cerebrovascular mortality (*p* for nonlinearity = 0.176). In contrast, the relationship between the AAPR and all-cause mortality followed an L-shaped pattern (*p* for nonlinearity < 0.001).

**Conclusions:**

The AAPR is important in predicting the risks associated with all-cause mortality and cardiovascular and cerebrovascular mortality, providing meaningful insights into mortality risk among the older adult population.

## 1. Introduction

Given the accelerating aging of the population, mortality attributable to cardiovascular and cerebrovascular diseases among older adults has increased [1]. Cardiovascular and cerebrovascular diseases—including heart failure, hypertension, coronary heart disease, and stroke—remain a major global health challenge and impose a substantial burden on healthcare systems [2, 3]. Although age, obesity, smoking, alcohol misuse, physical inactivity, cognitive dysfunction, and diabetes are well-documented risk factors for cardiovascular and cerebrovascular mortality, specific biomarkers that reliably predict these risks in older adults remain limited [4, 5, 6].

The albumin-to-alkaline phosphatase ratio (AAPR), an emerging and promising biomarker, is increasingly used in clinical practice and scientific research [7]. Accumulating evidence indicates that AAPR may serve as a valuable predictor of prognosis for several malignancies, including hepatocellular carcinoma, lung cancer, and renal cell carcinoma [8, 9, 10]. Albumin (ALB), primarily synthesized in the liver, is a significant component of serum proteins. It is crucial for maintaining physiological homeostasis and is frequently depleted during infection, inflammation, or systemic stress [11, 12]. Alkaline phosphatase (ALP) is a ubiquitous enzyme involved in cellular phosphorus metabolism and signal transduction pathways [13]. Clinically, ALP levels are commonly used in the diagnosis and monitoring of disorders affecting the skeletal and hepatobiliary systems.

Prior work has shown that the alkaline phosphatase–to–albumin ratio predicts prolonged harmful results of coronary artery disease with percutaneous coronary intervention (PCI) [14]. Furthermore, emerging data suggest that AAPR has superior predictive performance for cardiovascular mortality and fracture risk compared with traditional bone turnover markers, with higher AAPR levels correlating with improved survival [15, 16]. Notably, Li *et al*. [17] reported that preoperative AAPR combined with an inflammatory burden index effectively predicted overall survival in patients with rectal cancer.

Studies suggest that AAPR is a potential biomarker for cardiovascular and cerebrovascular mortality [18, 19]. However, evidence supporting this association remains insufficient. Given prior research on AAPR, its ease of accessibility, and computability, we hypothesized that it is related with all-cause and cardiovascular and cerebrovascular mortalities among the elderly people. Therefore, we investigated relationship between AAPR and cardiovascular and cerebrovascular prognosis in individuals aged 60 years and above, as well as potential underlying mechanisms, to provide insight into the utility of AAPR as a risk-stratification tool.

## 2. Materials and Method

### 2.1 Study Population

The National Health and Nutrition Examination Survey (NHANES), initiated in 1971, is a population-based program that assesses the health and nutritional status of children and adults in the United States and is conducted by trained professionals. Data were obtained from NHANES (2003–2018) via the official website (https://www.cdc.gov/nchs/nhanes). The study protocol was approved by the National Center for Health Statistics (NCHS) Ethics Review Board, and all participants gave consent. The study utilized publicly available data acquired through legal means and relied on anonymized information, fulfilling the criteria for exemption from ethical review. The Ethics Committee of Suining Central Hospital has granted this research an exemption, assigning it the ethics number KYLLMC20250032. This study was a retrospective analysis of NHANES data collected between 2003 and 2018. Inclusion criteria were adults aged 60 years and older. Exclusion criteria were age 0–59 years, missing data, or loss to follow-up. A total of 13,603 patients were recruited in the final analysis (**Supplementary Fig. 1**).

### 2.2 Data Collection

Baseline demographic and clinical data were obtained through NHANES household interviews, including gender, age, race, education level, marital status, body mass index (BMI), smoking status, alcohol consumption status, hypertension, diabetes, heart failure, coronary heart disease, myocardial infarction (MI), angina, arthritis, liver disease, thyroid disease, and tumor. Baseline laboratory tests included ALB, ALP, total protein, alanine aminotransferase (ALT), aspartate aminotransferase (AST), serum creatinine (Scr), uric acid (Ua), estimated glomerular filtration rate (eGFR), blood urea nitrogen (BUN), blood lipids, fasting plasma glucose, glycosylated hemoglobin (HbA1c), and globulin (GLB).

AAPR was calculated as ALB/ALP. Patients were ranked into quartiles (Q1–Q4) according to AAPR. With Q1 as the reference group, all-cause mortality and cardiovascular and cerebrovascular mortality for each quartile were estimated over the entire follow-up.

### 2.3 Assessment of Mortality

NHANES public-use mortality files through December 31, 2018, were used to determine participants’ death status via probabilistic matching with the National Death Index by the NCHS. The International Statistical Classification of Diseases and Related Health Problems, 10th Revision (ICD-10), was used to classify diseases, and cause-specific mortality was determined based on the NCHS classification of cardiovascular disease, cerebrovascular disease, and all other causes. Diagnoses of cardiovascular and cerebrovascular diseases were obtained from self-reported interviews using standardized medical condition questionnaires. Participants were asked: “Did your physician ever inform you about your hypertension, heart failure, coronary heart disease, MI, angina, or stroke?” Individuals were classified as having cardiovascular and cerebrovascular diseases if they responded ‘yes’ to any of these conditions.

### 2.4 Statistical Analysis

Data analysis was conducted using R 4.3.0 (Institute for Statistics and Mathematics, Vienna, Austria). Continuous variables are presented as mean ± standard deviation if normally distributed or median (IQR) otherwise. The Mann–Whitney U test was employed to compare quartiles, and chi-square tests were used to compare categorical variables. Multiple imputation addressed missing covariates with ≤15% missing values.

The Kaplan–Meier survival curves and the log-rank test were employed for comparison of all-cause and cardiovascular and cerebrovascular mortalities across AAPR quartiles. Three survey-weighted multivariate Cox proportional hazards models were constructed to evaluate the relationship between AAPR and all-cause mortality and cardiovascular and cerebrovascular mortality. Model 1 included no adjustments. Model 2 was tailored for gender, age, race, education level, marital status, poverty-income ratio (PIR), BMI, smoking status, and alcohol consumption; Model 3 additionally adjusted for eGFR, glycated hemoglobin (HbA1c), triglycerides (TG), high-density lipoprotein cholesterol (HDL-C), along with hypertension, diabetes, arthritis, heart failure, coronary artery disease, stroke, emphysema, liver disease, cancer, and renal failure. RCS was utilized to evaluate whether the relationship between AAPR and cardiovascular and cerebrovascular mortality was linear. Threshold effect analysis was conducted using a Cox proportional hazards regression model and a two-piecewise Cox proportional hazards regression model, with adjustment factors consistent with those described above.

Subgroup analyses were conducted by cardiovascular high-risk factors, including gender, race, BMI, smoking status, hypertension, diabetes, heart failure, coronary artery disease, stroke, emphysema, liver disease, and cancer. Deaths from non-cardiovascular and non-cerebrovascular causes were treated as competing events, and multivariate competing risk analysis (Fine-Gray model) was used to examine the relationship between AAPR and cardiovascular and cerebrovascular deaths. Mediation analysis was conducted to assess potential intermediary pathways linking AAPR with all-cause mortality and with cardiovascular and cerebrovascular mortality. A two-sided *p* < 0.05 was considered statistically significant.

## 3. Results

### 3.1 Demographic Characteristics of the Study Participants

**Supplementary Table 1** summarizes the demographic parameters of the 13,603 participants stratified by AAPR quartiles. The mean age was 70.48 ± 7.26 years; 49.81% were male, and the median AAPR was 0.592. Participants in higher AAPR groups tended to have lower body weight, were more probably non-Hispanic White, and were more often married. Smoking and alcohol consumption were associated with lower AAPR levels. Individuals with hypertension, heart failure, MI, angina, diabetes, stroke, arthritis, emphysema, chronic bronchitis, or liver disease were significantly underrepresented in the highest AAPR quartile than the lowest.

### 3.2 Analysis of the Association Between AAPR and All-cause Mortality and Cardiovascular and Cerebrovascular Mortality 

**Fig. 1.**
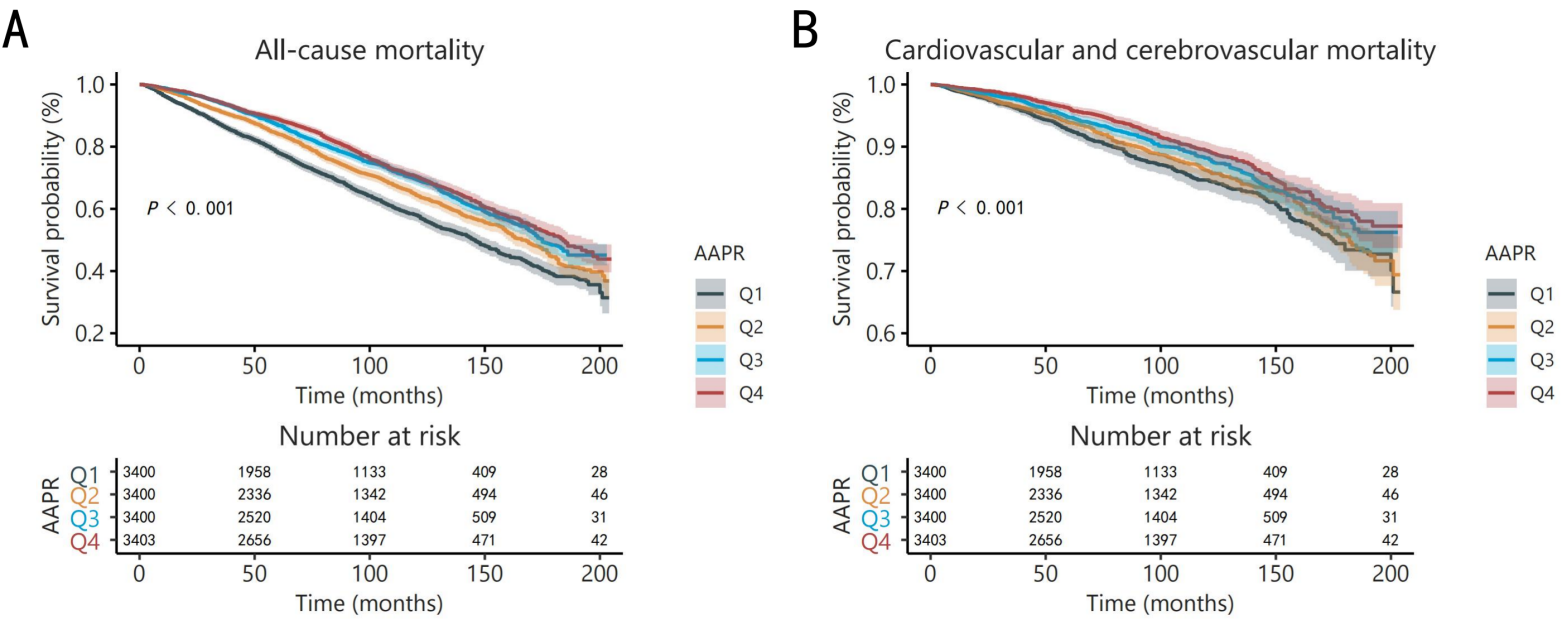
**Kaplan–Meier analyses for all-cause mortality (A) and cardiovascular and cerebrovascular mortality (B) among the AAPR quartiles**. Abbreviations: AAPR, albumin to alkaline phosphatase ratio.

During a median follow-up of 80 months (maximum 205 months), there were 4113 (30.24%) all-cause deaths and 1364 (10.03%) cardiovascular and cerebrovascular deaths. Kaplan–Meier analysis showed a much lower cumulative risk of both all-cause mortality and cardiovascular and cerebrovascular mortality among the highest AAPR quartile than with the lowest (Fig. 1).

**Fig. 2.**
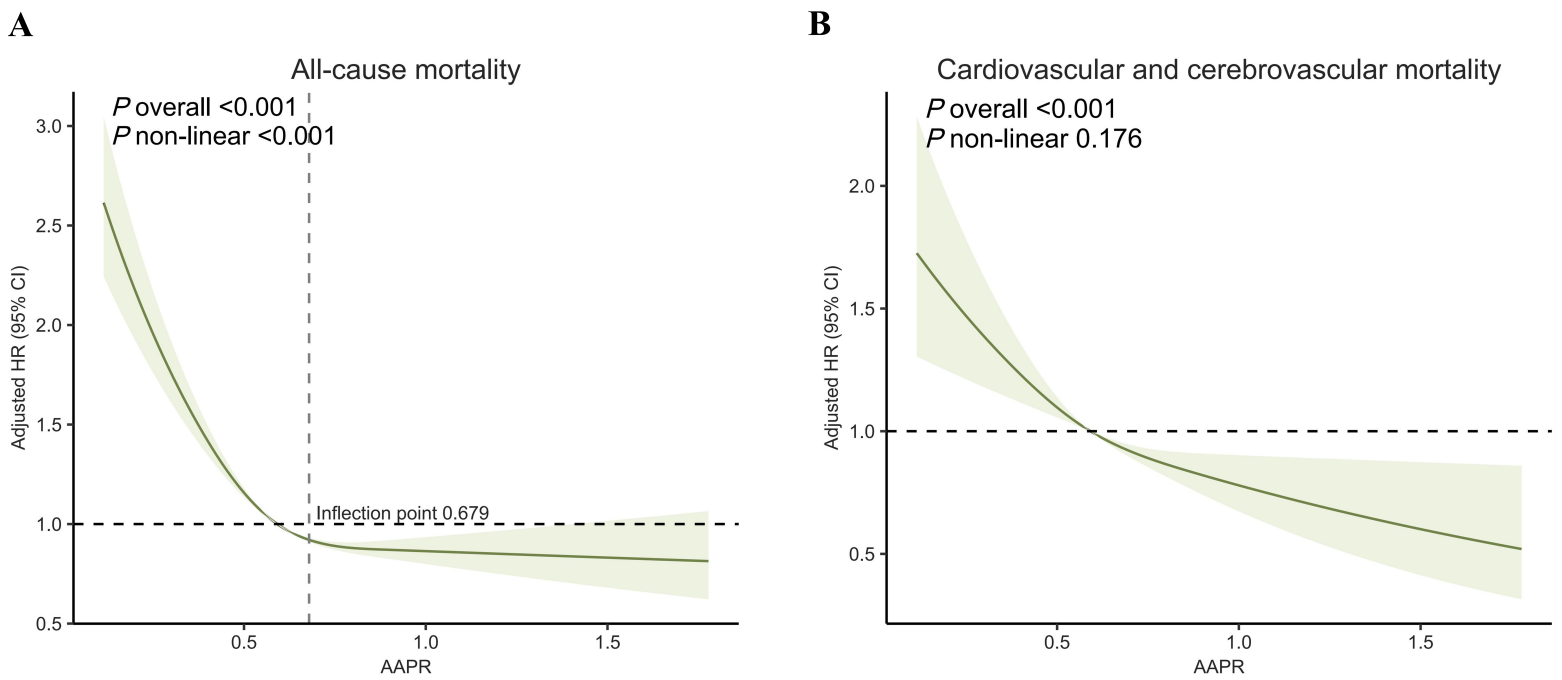
**Restricted cubic spline curves of AAPR for all-cause mortality (A) and cardiovascular-cerebrovascular mortality (B)**. Abbreviations: AAPR, albumin to alkaline phosphatase ratio; HR, hazard ratio; CI, confidence interval.

Trend regression analysis showed that higher AAPR was associated with reduced risk of all-cause mortality (HR = 0.59, 95% CI: 0.52–0.66, *p* for trend < 0.001 in Model 1; HR = 0.60, 95% CI: 0.53–0.67, *p* for trend < 0.001 in Model 2, and HR = 0.64, 95% CI: 0.57–0.71, *p* for trend < 0.001 in Model 3) and cardiovascular and cerebrovascular mortality (HR = 0.66, 95% CI: 0.54–0.80, *p* for trend < 0.001 in Model 1; HR = 0.69, 95% CI: 0.57–0.84, *p* for trend < 0.001 in Model 2, and HR = 0.73, 95% CI: 0.60–0.90, *p* for trend < 0.001 in Model 3) (Table 1). After adjusting the potential confounders, the smoothed curve fit indicated an L-shaped association between AAPR and the risk of all-cause mortality (*p* for nonlinearity < 0.001), whereas a negative linear relationship was observed between AAPR and the risk of cardiovascular and cerebrovascular mortality (*p* for nonlinearity = 0.176) (Fig. 2). We also implemented RCS based on the Cox regression model in men and women aged ≥60 years. The results showed L-shaped associations between AAPR and all-cause mortality risk in both sexes (both *p* for nonlinearity < 0.001) and negative linear relationships with cardiovascular and cerebrovascular mortality risk (*p* for nonlinearity = 0.112; *p* for nonlinearity = 0.874) (**Supplementary Fig. 2**).

**Table 1.** **HRs (95% CIs) for mortality according to the AAPR quartiles**.

Variables			Overall	Quartiles of AAPR				*p* for trend
			Per 0.1 unit	Q1 (0.022–0.474)	Q2 (0.474–0.592)	Q3 (0.592–0.729)	Q4 (0.729–3.143)	
All-cause mortality								
Number of deaths			N = 4113	N = 1186	N = 1076	N = 951	N = 900	
	Model 1							<0.001
		HR (95% CI)	0.91 (0.89, 0.93)	1 (Ref)	0.79 (0.71, 0.88)	0.62 (0.56, 0.69)	0.59 (0.52, 0.66)	
		*p* value	<0.001		<0.001	<0.001	<0.001	
	Model 2							<0.001
		HR (95% CI)	0.91 (0.89, 0.93)	1 (Ref)	0.80 (0.72, 0.89)	0.62 (0.55, 0.69)	0.60 (0.53, 0.67)	
		*p* value	<0.001		<0.001	<0.001	<0.001	
	Model 3							<0.001
		HR (95% CI)	0.92 (0.90, 0.94)	1 (Ref)	0.86 (0.77, 0.96)	0.66 (0.59, 0.74)	0.64 (0.57, 0.71)	
		*p* value	<0.001		0.005	<0.001	<0.001	
Cardiovascular mortality								
Number of deaths			N = 1364	N = 362	N = 373	N = 333	N = 296	
	Model 1							<0.001
		HR (95% CI)	0.93 (0.89, 0.96)	1 (Ref)	0.92 (0.77, 1.10)	0.75 (0.62, 0.90)	0.66 (0.54, 0.80)	
		*p* value	<0.001		0.359	0.002	<0.001	
	Model 2							<0.001
		HR (95% CI)	0.93 (0.90, 0.97)	1 (Ref)	0.94 (0.79, 1.13)	0.76 (0.63, 0.91)	0.69 (0.57, 0.84)	
		*p* value	<0.001		0.528	0.003	<0.001	
	Model 3							<0.001
		HR (95% CI)	0.94 (0.90, 0.97)	1 (Ref)	1.04 (0.87, 1.24)	0.81 (0.68, 0.98)	0.73 (0.60, 0.90)	
		*p* value	<0.001		0.705	0.03	0.002	

Model 1: Unadjusted. Model 2: Adjusted for gender, age, race, education level, marital status, PIR, BMI, smoking status, and alcohol consumption status. Model 3: Further adjusted for eGFR, HbA1c, TG, HDL-C, hypertension, diabetes, arthritis, heart failure, coronary heart diseases, stroke, emphysema, liver diseases, cancer, and renal failure. Abbreviations: AAPR, albumin to alkaline phosphatase ratio; Ref, reference; BMI, body mass index; PIR, poverty impact ratio; HbA1c, glycosylated hemoglobin; eGFR, estimated glomerular filtration rate; TG, triglyceride; HDL-C, high-density lipoprotein cholesterol; HR, hazard ratio; CI, confidence interval.

### 3.3 Analysis of Threshold Effect

Cox proportional hazards regression models and two-piecewise Cox proportional hazards regression models were utilized to examine the link between AAPR and all-cause mortality. Analyses were conducted in the total population and separately for males and females to assess gender-specific patterns. Inflection points of 0.679, 0.728, and 0.652 were identified for all-cause mortality in the total population, male subgroup, and female subgroup, respectively (all *p* values for log-likelihood ratio <0.001). For each 0.1-unit increase in AAPR up to the inflection points, the risk of all-cause mortality decreased by 16%, 17%, and 16% in the total population, male subgroup, and female subgroup, respectively. Notably, once AAPR reached the respective inflection point, the risk of all-cause mortality declined to its lowest level and subsequently exhibited a plateau effect, indicating no further statistically significant reduction in mortality risk with increasing AAPR (all *p* > 0.05) (Table 2).

**Table 2.** **Threshold effect analysis of AAPR on all-cause mortality**.

Variables		Adjusted HR (95% CI), *p* value
All-cause mortality		
	Model 1 standard line regression	0.92 (0.91, 0.94), *p * < 0.001
	Model 2 two-piece wise linear regression	
	Inflection point	0.679
	AAPR <0.679	0.84 (0.81, 0.87), *p * < 0.001
	AAPR ≥0.679	1.00 (0.97, 1.03), *p* = 0.993
	Log-likelihood ratio test	*p * < 0.001
All-cause mortality for male		
	Model 1 standard line regression	0.91 (0.88, 0.93), *p * < 0.001
	Model 2 two-piece wise linear regression	
	Inflection point	0.728
	AAPR <0.728	0.84 (0.80, 0.88), *p * < 0.001
	AAPR ≥0.728	1.00 (0.95, 1.05), *p* = 1.000
	Log-likelihood ratio test	*p * <0.001
All-cause mortality for female		
	Model 1 standard line regression	0.93 (0.90, 0.95), *p * < 0.001
	Model 2 two-piece wise linear regression	
	Inflection point	0.652
	AAPR <0.652	0.84 (0.80, 0.89), *p * < 0.001
	AAPR ≥0.652	1.00 (0.96, 1.04), *p* = 0.993
	Log-likelihood ratio test	*p * < 0.001

We fit Cox proportional hazards regression models to assess hazard ratios (HRs) and 95% confidence intervals (CIs). Models were adjusted for sex, age, race, educational attainment, marital status, PIR, BMI, smoking status, alcohol consumption, eGFR, HbA1c, TG, HDL-C, hypertension, diabetes, arthritis, heart failure, coronary heart disease, stroke, emphysema, liver disease, cancer, and renal failure. Abbreviations: AAPR, albumin to alkaline phosphatase ratio; PIR, poverty–income ratio; BMI, body mass index; eGFR, estimated glomerular filtration rate; HbA1c, glycosylated hemoglobin; TG, triglycerides; HDL-C, high-density lipoprotein cholesterol.

### 3.4 Subgroup Analysis

**Fig. 3.**
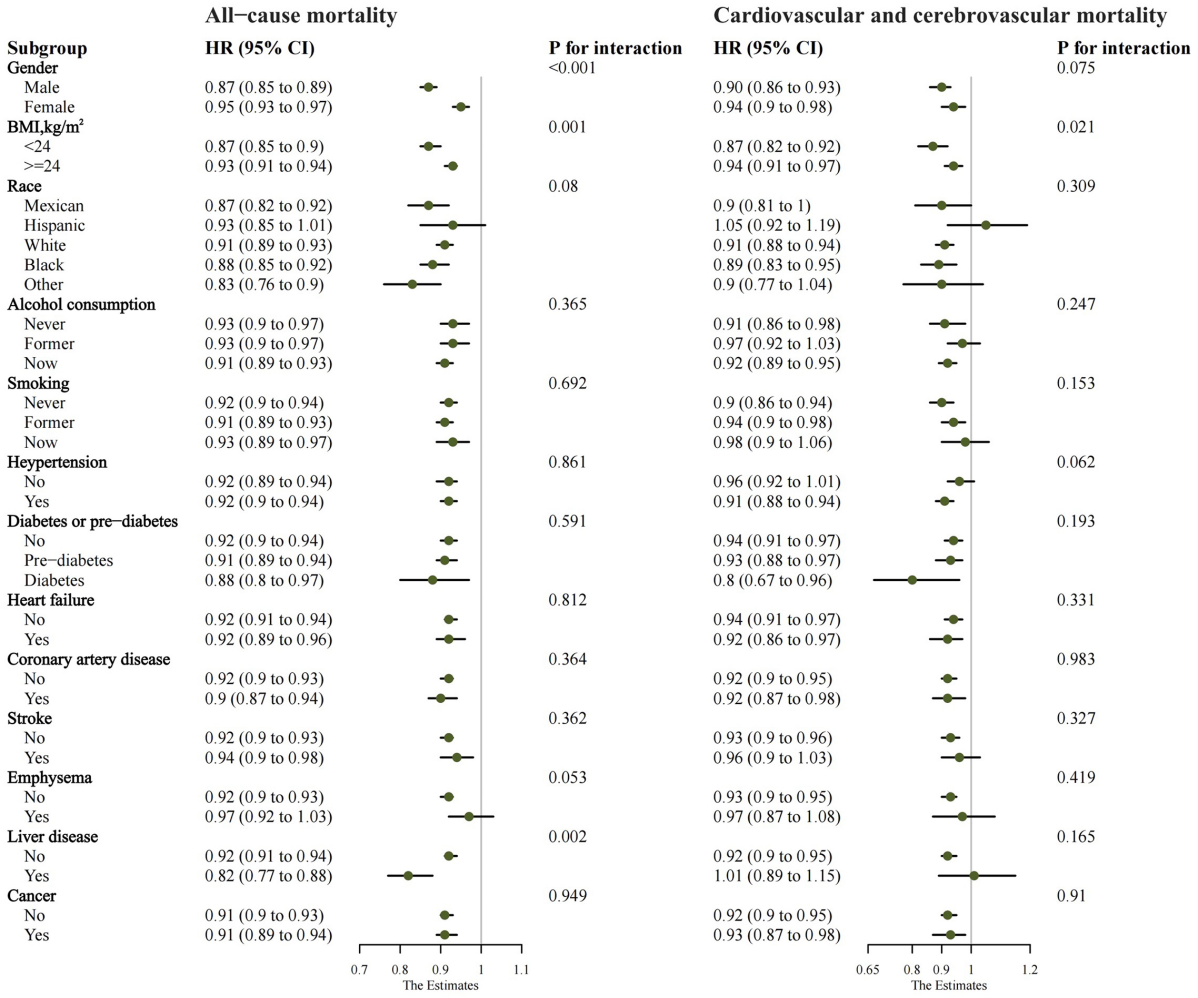
**Subgroup analysis of AAPR for all-cause mortality and cardiovascular and cerebrovascular mortality**. Abbreviations: AAPR, albumin to alkaline phosphatase ratio; BMI, body mass index; HR, hazard ratio; CI, confidence interval.

Subgroup analysis showed no important interactions between AAPR and race, alcohol use, smoking, hypertension, diabetes, heart failure, coronary artery disease, stroke, emphysema, liver disease, or cancer for all-cause mortality (*p* for interaction > 0.05), showing the consistent predictive value of AAPR across these subgroups. Significant interactions were observed with gender, BMI, and liver disease (*p* for interaction < 0.05). For cardiovascular and cerebrovascular mortality, no significant interactions were observed by gender, BMI, race, alcohol use, smoking, hypertension, diabetes, heart failure, coronary artery disease, stroke, emphysema, liver disease, or cancer (*p *for interaction > 0.05), indicating consistent AAPR prediction. However, a significant interaction was detected for the BMI subgroup (*p* for interaction < 0.05) (Fig. 3).

### 3.5 Sensitivity Analysis of AAPR and Cardiovascular and Cerebrovascular Mortality 

The sensitivity of AAPR for predicting cardiovascular and cerebrovascular mortality was further evaluated by incorporating AAPR, ALB, ALP, and risk factors associated with cardiovascular and cerebrovascular mortality into multivariate competing-risk models, respectively. AAPR was an independent risk factor for cardiovascular and cerebrovascular mortality (HR = 0.96, 95% CI: 0.93–0.99, *p* = 0.005) (**Supplementary Fig. 3**). In contrast, ALB (HR = 0.93, 95% CI: 0.76–1.15, *p* = 0.52) and ALP (HR = 1.00, 95% CI: 1.00–1.00, *p* = 0.081) were not statistically significant, indicating that ALB and ALP were not independent risk factors for cardiovascular and cerebrovascular mortality (**Supplementary Figs. 4,5**).

### 3.6 Mediation Analysis

**Fig. 4.**
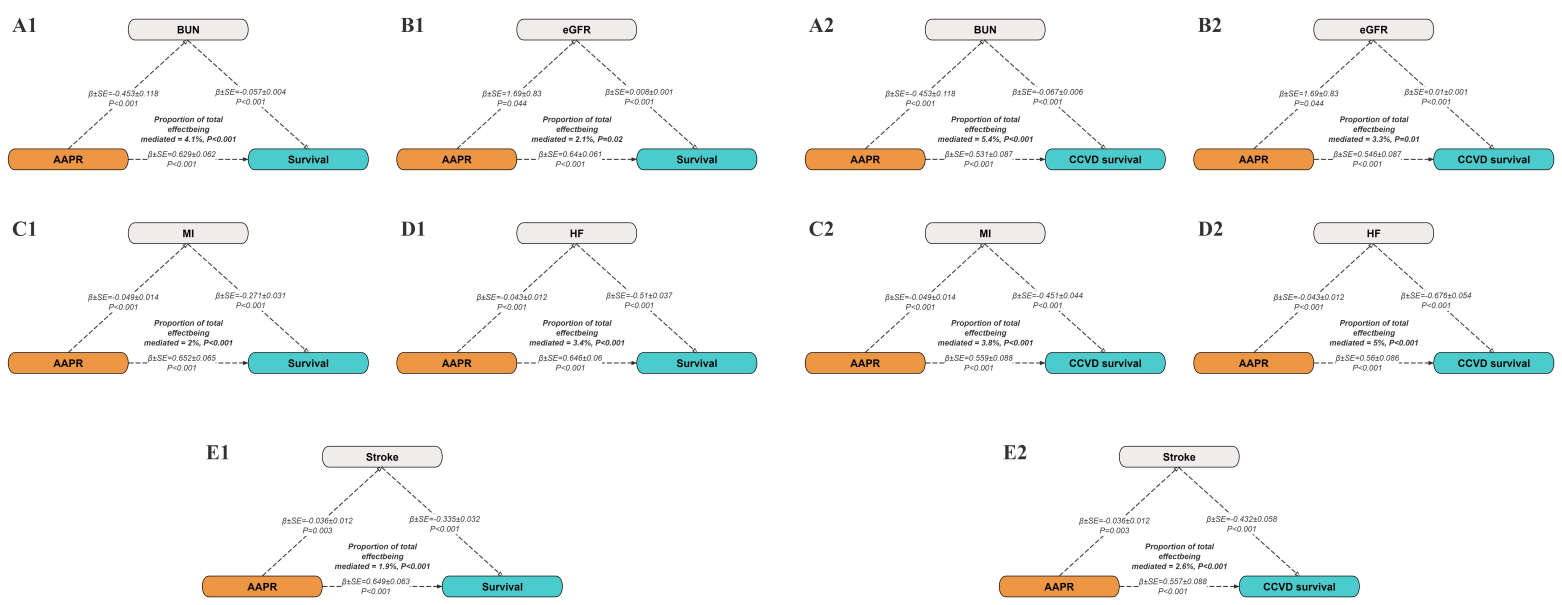
**Mediation analysis of associations between AAPR and all-cause mortality and cardiovascular and cerebrovascular mortality**. A1–E1 present the mediating effects of BUN, eGFR, MI, HF and stroke on survival rate as mediating variables. A2–E2 present the mediating effects of the same mediating variables on the survival rate of cardiovascular and cerebrovascular diseases. Each model includes coefficients (β), standard errors (SE), *p* values and the proportion of the contribution of the mediating variables to the total effect. Abbreviations: AAPR, albumin to alkaline phosphatase ratio; CCVD, cardiovascular and cerebrovascular disease; BUN, blood urea nitrogen; eGFR, estimated glomerular filtration rate; MI, myocardial infarction; HF, heart failure.

Mediation analysis included BUN, eGFR, MI, heart failure, and stroke to examine their potential roles in the associations between AAPR and mortality outcomes. BUN accounted for 4.1% of the relation between AAPR and all-cause mortality and 5.4% of the association with cardiovascular and cerebrovascular mortality. eGFR accounted for 2.1% and 3.3% of the total effect on all-cause and cardiovascular and cerebrovascular mortality, respectively. In addition, AAPR was significantly and negatively related with MI, heart failure, and stroke (all *p* < 0.001), each of which was independently related to increased mortality risk. These factors mediated the following proportions of the total effect: MI (2.0% for all-cause and 3.8% for cardiovascular and cerebrovascular mortality), heart failure (3.4% and 5.0%), and stroke (1.9% and 2.6%). Collectively, these findings highlight the role of AAPR in influencing mortality through pathways related to renal function and cardiovascular health (Fig. 4).

## 4. Discussion

AAPR, a novel biomarker, is a prognostic factor for many malignancies [20, 21, 22]. Previous studies indicate that AAPR is related to bone metabolism and serves as a prognostic biomarker for spinal fusion in patients with lumbar degenerative disease undergoing lumbar spinal fusion [16, 23]. Mathold *et al*. [15] reported that AAPR influences the prognosis of cardiovascular diseases. However, few studies have systematically assessed the association between AAPR and cardiovascular and cerebrovascular mortality.

No prior literature, to our knowledge, has specifically evaluated the relation between AAPR and all-cause mortality and cardiovascular and cerebrovascular mortality among adults aged 60 years and above, indicating that AAPR is a robust predictor of these outcomes. A negative linear relationship was observed between AAPR and cardiovascular and cerebrovascular mortality (*p* for nonlinearity = 0.176), suggesting that cardiovascular and cerebrovascular mortality risk consistently decreases with increasing AAPR levels.

Conversely, the link between AAPR and all-cause mortality exhibited an L-shaped curve (*p* for nonlinearity < 0.001). Before the inflection point, the risk of all-cause mortality declined by 16% with every 0.1-unit increase in AAPR. Beyond this threshold, a plateau effect was observed, with no further significant decline in mortality risk despite continued increases in AAPR. These findings were consistently replicated in both male and female subgroups.

Subgroup analysis showed no statistically significant relations between AAPR and most subgroups. However, the interaction effect of AAPR was stronger in males, individuals with lower BMI (<24 kg/m^2^), and patients with liver disease. Regarding gender differences, compared with men, women typically have higher baseline albumin levels (reflecting differences in muscle mass and hormone regulation) and lower alkaline phosphatase levels (reduced bone turnover in women due to postmenopausal hormonal changes) [24, 25]. Alterations in hepatic protein synthesis that reduce albumin are more common in individuals with obesity; thus, AAPR may serve as a more sensitive marker of systemic dysregulation in this population, enhancing its prognostic value [26]. In patients with liver disease, hepatic dysfunction directly impairs albumin synthesis (reducing albumin) and disrupts bile acid metabolism (increasing ALP), making AAPR a direct indicator of liver function in this subgroup [27]. Sensitivity analysis showed that AAPR was an independent risk factor for cardiovascular mortality (*p* = 0.005), and AAPR was more efficient in predicting all-cause mortality and cardiovascular and cerebrovascular mortality compared with ALB and ALP. We further examined the link of AAPR with all-cause mortality and cardiovascular and cerebrovascular mortality by mediation analysis and found that AAPR influenced these outcomes through effects on BUN, eGFR, MI, heart failure, and stroke. The data originated from the NHANES database (2003–2018), and 13,603 cases were included. Therefore, we believe that applying AAPR to predict the prognosis of all-cause and cardiovascular and cerebrovascular mortality in adults aged 60 years and above is highly credible.

AAPR, the ratio of ALB to ALP, plays a crucial role in cardiovascular and cerebrovascular mortality. On one hand, ALB, the most abundant protein in blood, has multiple physiological functions to maintain osmotic pressure and is involved in oxidative stress responses, anticoagulation, and antiplatelet aggregation [28, 29, 30]. Low ALB reflects malnutrition, liver dysfunction, or inflammatory status [11, 31]. Low ALB reduces fibrinolytic and antioxidant capacities, damages endothelial cells, activates inflammatory responses, and increases the risk of thrombosis, leading to adverse cardiovascular and cerebrovascular events [32, 33]. Some studies suggest that low ALB is significantly related with poor long-term prognosis in patients undergoing PCI, and that preoperative ALB can serve as a clinical predictor of future cardiovascular events in patients with stable coronary heart disease and preserved renal function [34].

On the other hand, ALP is an enzyme widely present in many tissues and is associated with pathological processes, including inflammation, metabolic disorders, and atherosclerosis [35, 36, 37]. High ALP may promote inflammation, affect mineral metabolism, and accelerate atherosclerosis, ultimately leading to cardiovascular and cerebrovascular atherosclerosis [38]. Additionally, Dai *et al*. [14] reported that ALP was associated with mortality in coronary heart disease, recurrent infarction, and thromboembolic events after PCI. Wang *et al*. [39] found that ALP was associated with slow flow in patients with coronary heart disease; patients with high ALP were more susceptible to coronary slow flow. High ALP also affected pyrophosphate metabolism, promoted vascular calcification, compromised vascular integrity, and worsened atherosclerosis [40, 41]. Erez *et al*. [42] reported that ALP, combined with intracranial artery calcification, served as a prognostic predictor in patients with nephropathy undergoing hemodialysis, suggesting that high ALP levels were associated with mortality in chronic kidney disease. Haarhaus *et al*. [43] characterized ALP as a clinically actionable target for cardiovascular and mineral–bone pathology in the context of chronic kidney disease. Therefore, AAPR comprehensively reflects the levels of albumin and alkaline phosphatase. Low AAPR may indicate a pathological condition associated with aging-related chronic diseases such as heart failure, liver disease, and renal failure, and suggests that chronic inflammation inhibits albumin synthesis, promotes ALP release, and accelerates vascular endothelial injury and atherosclerosis. Consequently, AAPR may serve as a novel prognostic indicator for patients with cardiovascular and cerebrovascular diseases. Clinicians should assess AAPR alongside the overall clinical condition of the patient and rule out reversible factors affecting ALB or ALP to avoid reliance on a single indicator.

## 5. Strengths and Limitations

The study findings suggest that AAPR can be applied in clinical practice. First, as an easily obtainable prognostic indicator for routine health evaluations and chronic disease management in older adults—derived from standard laboratory tests—it enables risk stratification. Second, as an adjunct to existing risk tools, it can capture concealed nutritional impairments and mild inflammation that traditional scores may overlook, thereby improving predictive accuracy.

One constraint of this study is the reliance on a single initial assessment of AAPR to forecast long-term mortality outcomes. Biological markers such as ALB and ALP can vary over time—for example, with changes in nutritional status or inflammatory processes—and a solitary measurement may not reflect the overall health. A further limitation involves the intrinsic selection bias associated with the NHANES database. Nonresponse bias remains a concern, as individuals who choose to participate are typically more attentive to health, including being more likely to follow medical advice and provide accurate accounts of health-related behaviors, than those who decline participation. Despite comprehensive adjustments for factors influencing outcomes, residual confounding may persist, including genetic predispositions and underreported comorbidities.

Therefore, multicenter, large-scale studies must be performed to further confirm the prognostic value of AAPR in cardiovascular and cerebrovascular diseases, with endpoint events that include MI, heart failure, arrhythmia, stroke, hemorrhagic stroke, and transient ischemic attack. AAPR should be dynamically monitored in accordance with the health status of older adults during routine physical examinations or hospitalizations at community healthcare facilities. Further research must aim to reveal the mechanisms linking AAPR to cardiovascular and cerebrovascular diseases.

## 6. Conclusions

AAPR effectively predicts the risks of all-cause mortality and cardiovascular and cerebrovascular mortality in individuals older than 60 years. It could act as a novel prognostic indicator in this population. Clinicians may utilize AAPR to inform treatment planning and follow-up for older patients, thereby improving quality of life.

## Availability of Data and Materials

The datasets that were used and evaluated in this study can be obtained from the corresponding author upon making a reasonable request.

## Supplementary Material

Supplementary material associated with this article can be found, in the online version, at https://doi.org/10.31083/RCM45049.
