# Supplementary material for: Employment of the Albumin to Alkaline Phosphatase Ratio to Predict All-cause Mortality and Cardiovascular and Cerebrovascular Mortality in Adults Aged At Least 60 Years: An Analysis From NHANES 2003–2018
Source: Rev Cardiovasc Med. 2026 Feb 6;27(2):45049. doi: 10.31083/RCM45049 (PMC12959993; doi:10.31083/RCM45049)
Supplement: Supplementary file 1 [file 2153-8174-27-2-45049-s1.zip › Supplementary Material.doc]

Supplementary Fig. 1 [Flow chart of study participants](http://fanyi.sogou.com/?keyword=flow chart of study participants&from=en&to=zh-CHS&fr=websearch)

Supplementary Fig. 2 The restricted cubic spline curves of AAPR for all-cause mortality for male (A) and female (B). The restricted cubic spline curves of AAPR for cardiovascular and cerebrovascular mortality for male (C) and female (D). Abbreviations: AAPR: albumin to alkaline phosphatase ratio

Supplementary Fig. 3 Multivariate competitive risk analysis of AAPR for cardiovascular and cerebrovascular mortality. Abbreviations: AAPR: albumin to alkaline phosphatase ratio; BMI: body mass index; PIR: poverty impact ratio; AST: aspartate aminotransferase; TP: total protein; GLB: globulin; Scr: serum creatinine; Ua: uric acid; eGFR: estimated glomerular filtration rate; BUN: blood urea nitrogen; TC: total cholesterol; TG: triglyceride; HDL-C: high-density lipoprotein cholesterol; LDL-C: low-density lipoprotein cholesterol; FBG: fasting blood glucose; HbA1c: glycosylated hemoglobin; MI: myocardial infarction

Supplementary Fig. 4 Multivariate competitive risk analysis of ALB for cardiovascular and cerebrovascular mortality. Abbreviations: ALP: alkaline phosphatase; BMI: body mass index; PIR: poverty impact ratio; AST: aspartate aminotransferase; TP: total protein; GLB: globulin; UA: uric acid; BUN: blood urea nitrogen; Scr: serum creatinine; eGFR: estimated glomerular filtration rate; TC: total cholesterol; TG: triglyceride; HDL-C: high-density lipoprotein cholesterol; LDL-C: low-density lipoprotein cholesterol; FPG: fasting plasma glucose; HbA1c: glycosylated hemoglobin; MI: myocardial infarction

Supplementary Fig. 5 Multivariate competitive risk analysis of ALP for cardiovascular and cerebrovascular mortality. Abbreviations: ALP: alkaline phosphatase; BMI: body mass index; PIR: poverty impact ratio; AST: aspartate aminotransferase; TP: total protein; GLB: globulin; UA: uric acid; BUN: blood urea nitrogen; Scr: serum creatinine; eGFR: estimated glomerular filtration rate; TC: total cholesterol; TG: triglyceride; HDL-C: high-density lipoprotein cholesterol; LDL-C: low-density lipoprotein cholesterol; FPG: fasting plasma glucose; HbA1c: glycosylated hemoglobin; MI: myocardial infarction

| **Supplementary Table. 1 Demographic characteristics of the study participants** | | | | | | |
| --- | --- | --- | --- | --- | --- | --- |
| Characteristic | Total | AAPR | | | | *P* |
|  |  | Q1(0.022-0.474) Q2(0.474-0.592) Q3(0.592-0.729) Q4(0.729-3.143) | | | |  |
| Participants | n=13603 | n=3402 | n=3427 | n=3396 | n=3378 |  |
| Age, years | 70.48±7.26 | 70.22±7.37 | 70.29±7.31 | 70.63±7.22 | 70.79±7.13 | 0.003 |
| Gender, male, n (%) | 6775 (49.81) | 1490 (43.80) | 1586 (46.28) | 1815 (53.45) | 1884 (55.77) | <0.001 |
| BMI, kg/m2 | 29.02±6.15 | 29.89±6.92 | 29.26±6.12 | 28.89±5.80 | 28.06±5.55 | <0.001 |
| Time, month | 86.50±51.87 | 76.24±53.90 | 86.55±52.73 | 90.75±50.88 | 92.53±48.24 | <0.001 |
| PIR | 2.50±1.54 | 2.23±1.47 | 2.42±1.52 | 2.56±1.55 | 2.79±1.57 | <0.001 |
| Education level, n (%) |  |  |  |  |  | <0.001 |
| Less than high school | 3241 (23.88) | 799 (23.58) | 791 (23.10) | 824 (24.32) | 827 (24.54) |  |
| High school | 4366 (32.17) | 1290 (38.08) | 1229 (35.89) | 1001 (29.55) | 846 (25.10) |  |
| College or above | 5963 (43.94) | 1299 (38.34) | 1404 (41.00) | 1563 (46.13) | 1697 (50.36) |  |
| Race, n (%) |  |  |  |  |  | <0.001 |
| Mexican | 1862 (13.69) | 596 (17.52) | 537 (15.67) | 428 (12.60) | 301 (8.91) |  |
| Hispanic | 1138 (8.37) | 325 (9.55) | 294 (8.58) | 298 (8.78) | 221 (6.54) |  |
| Non-Hispanic White | 6935 (50.98) | 1468 (43.15) | 1694 (49.43) | 1805 (53.15) | 1968 (58.26) |  |
| Non-Hispanic Black | 2648 (19.47) | 809 (23.78) | 679 (19.81) | 614 (18.08) | 546 (16.16) |  |
| Other Race | 1020 (7.50) | 204 (6.00) | 223 (6.51) | 251 (7.39) | 342 (10.12) |  |
| Smoking status, n (%) |  |  |  |  |  | <0.001 |
| Never | 6587 (48.49) | 1570 (46.24) | 1712 (50.00) | 1645 (48.50) | 1660 (49.20) |  |
| Former | 5263 (38.74) | 1232 (36.29) | 1234 (36.04) | 1364 (40.21) | 1433 (42.47) |  |
| Now | 1735 (12.77) | 593 (17.47) | 478 (13.96) | 383 (11.29) | 281 (8.33) |  |
| Alcohol consumption status, n (%) |  |  |  |  |  | <0.001 |
| Never | 2215 (17.38) | 597 (18.99) | 604 (18.84) | 537 (16.65) | 477 (15.03) |  |
| Former | 2499 (19.60) | 754 (23.99) | 654 (20.40) | 575 (17.83) | 516 (16.26) |  |
| Now | 8034 (63.02) | 1792 (57.02) | 1948 (60.76) | 2113 (65.52) | 2181 (68.71) |  |
| Marriage |  |  |  |  |  | <0.001 |
| Married or living with a partner, n (%) | 7843 (57.69) | 1786 (52.56) | 1923 (56.13) | 2003 (59.03) | 2131 (63.10) |  |
| Widowhood or divorce | 5082 (37.38) | 1416 (41.67) | 1337 (39.03) | 1238 (36.49) | 1091 (32.31) |  |
| Unmarried | 669 (4.92) | 196 (5.77) | 166 (4.85) | 152 (4.48) | 155 (4.59) |  |
| Comorbidity |  |  |  |  |  |  |
| Hypertention, n (%) | 8243 (60.74) | 2158 (63.62) | 2053 (60.08) | 2017 (59.55) | 2015 (59.70) | 0.001 |
| Pre-Diabetes, n (%) | 470 (3.46) | 94 (2.76) | 121 (3.53) | 136 (4.01) | 119 (3.53) | <0.001 |
| Diabetes, n (%) | 3230 (23.76) | 990 (29.12) | 742 (21.68) | 703 (20.71) | 795 (23.56) | <0.001 |
| Heart failure, n (%) | 1040 (7.70) | 353 (10.45) | 248 (7.29) | 226 (6.70) | 213 (6.33) | <0.001 |
| Coronary heart disease, n (%) | 1392 (10.33) | 377 (11.18) | 332 (9.78) | 335 (9.95) | 348 (10.39) | 0.233 |
| MI, n (%) | 1356 (10.00) | 411 (12.13) | 325 (9.51) | 312 (9.21) | 308 (9.14) | <0.001 |
| Angina, n (%) | 824 (6.10) | 242 (7.16) | 214 (6.30) | 180 (5.33) | 188 (5.60) | 0.008 |
| Stroke, n (%) | 1171 (8.63) | 357 (10.52) | 288 (8.43) | 280 (8.26) | 246 (7.29) | <0.001 |
| Arthritis, n (%) | 6839 (50.44) | 1777 (52.42) | 1781 (52.15) | 1669 (49.31) | 1612 (47.85) | <0.001 |
| Emphysema, n (%) | 589 (4.34) | 162 (4.78) | 172 (5.03) | 138 (4.07) | 117 (3.47) | <0.001 |
| Chronic bronchitis, n (%) | 1054 (7.78) | 326 (9.61) | 274 (8.03) | 235 (6.94) | 219 (6.50) | 0.007 |
| Liver diseases, n (%) | 686 (5.06) | 245 (7.23) | 162 (4.74) | 133 (3.93) | 146 (4.33) | <0.001 |
| Thyroid disease, n (%) | 2306 (16.99) | 610 (17.97) | 562 (16.44) | 599 (17.69) | 535 (15.88) | 0.066 |
| Renal failure, n(%) | 780 (5.75) | 320 (9.44) | 178 (5.21) | 135 (3.98) | 147 (4.36) | <0.001 |
| Cancer, n (%) | 2682 (19.74) | 659 (19.38) | 654 (19.10) | 650 (19.17) | 719 (21.33) | 0.065 |
| Laboratory test |  |  |  |  |  |  |
| ALB, g/L | 41.42±3.25 | 39.45±3.45 | 41.20±2.90 | 42.09±2.78 | 42.96±2.76 | <0.001 |
| ALP, U/L | 74.74±29.79 | 107.80±39.39 | 77.46±7.09 | 64.34±5.48 | 49.15±7.59 | <0.001 |
| AAPR | 0.62±0.21 | 0.39±0.07 | 0.53±0.03 | 0.66±0.04 | 0.90±0.18 | <0.001 |
| TP, g/L | 70.94±4.95 | 71.05±5.33 | 71.00±5.03 | 70.90±4.80 | 70.80±4.61 | 0.178 |
| AST, U/L | 25.26±18.50 | 26.51±32.70 | 24.62±10.45 | 24.77±9.85 | 25.16±9.56 | <0.001 |
| ALT, U/L | 22.35±22.34 | 23.42±39.84 | 21.85±11.93 | 21.85±11.96 | 22.31±11.04 | 0.011 |
| Scr, mg/dL | 1.021±0.55 | 1.09±0.83 | 0.99±0.47 | 0.99±0.36 | 1.01±0.42 | <0.001 |
| eGFR, ml/min | 77.28±29.40 | 77.46±32.46 | 78.64±29.28 | 77.62±28.06 | 75.38±27.54 | <0.001 |
| Ua, umol/L | 340.54±88.29 | 339.37±93.66 | 338.15±88.27 | 344.37±86.58 | 340.27±84.27 | 0.023 |
| BUN, umol/L | 6.00±2.69 | 6.26±3.28 | 5.87±2.61 | 5.86±2.26 | 6.02±2.46 | <0.001 |
| TC, mmol/L | 5.01±1.15 | 4.92±1.20 | 5.08±1.15 | 5.06±1.15 | 4.99±1.11 | <0.001 |
| TG, mmol/L | 1.75±1.18 | 1.76±1.32 | 1.78±1.12 | 1.75±1.18 | 1.69±1.10 | 0.017 |
| LDL-C, mmol/L | 2.88±0.97 | 2.83±1.02 | 2.94±0.98 | 2.91±0.97 | 2.83±0.92 | 0.001 |
| HDL-C, mmol/L | 1.41±0.43 | 1.38±0.42 | 1.40±0.41 | 1.41±0.41 | 1.45±0.47 | <0.001 |
| FPG, mmol/L | 6.57±2.24 | 6.97±2.94 | 6.51±2.11 | 6.44±1.95 | 6.33±1.68 | <0.001 |
| Insulin, UI | 14.09±18.95 | 15.62±24.09 | 14.08±17.68 | 13.27±13.34 | 13.36±18.91 | 0.001 |
| HbA1c, mmol/L | 6.07±1.13 | 6.31±1.46 | 6.06±1.09 | 5.98±0.94 | 5.95±0.88 | <0.001 |
| GLB, g/L | 29.52±5.00 | 31.60±5.42 | 29.80±4.87 | 28.82±4.58 | 27.86±4.26 | <0.001 |
| All-cause mortality, n (%) | 4113 (30.24) | 1186 (34.86) | 1076 (31.40) | 951 (28.00) | 900 (26.64) | <0.001 |
| Cardiovascular and cerebrovascular mortality, n (%) | 1364 (10.03) | 362 (10.64) | 373 (10.88) | 333 (9.81) | 296 (8.76) | 0.016 |

Abbreviations: AAPR: albumin to alkaline phosphatase ratio; BMI: body mass index; PIR: poverty impact ratio; MI: myocardial infarction; ALB: albumin; ALP: Alkaline phospholipase; ALT: alanine aminotransferase; AST: aspartate aminotransferase; TP: total protein; Scr: serum creatinine; Ua: uric acid; eGFR: estimated glomerular filtration rate; BUN: blood urea nitrogen; TC: total cholesterol; TG: triglyceride; HDL-C: high-density lipoprotein cholesterol; LDL-C: low-density lipoprotein cholesterol; FPG: fasting plasma glucose; HbA1c: glycosylated hemoglobin; GLB: globulin
